# Activation of TGR5 Partially Alleviates High Glucose-Induced Cardiomyocyte Injury by Inhibition of Inflammatory Responses and Oxidative Stress

**DOI:** 10.1155/2019/6372786

**Published:** 2019-11-21

**Authors:** Li Deng, Xuxin Chen, Yi Zhong, Xing Wen, Ying Cai, Jiafu Li, Zhongcai Fan, Jian Feng

**Affiliations:** ^1^Department of Cardiology, The Affiliated Hospital of Southwest Medical University, Luzhou, Sichuan, China; ^2^Department of Rheumatology, The Affiliated Hospital of Southwest Medical University, Luzhou, Sichuan, China; ^3^Department of Pulmonary and Critical Care Medicine, The Sixth Medical Center of Chinese PLA General Hospital, Beijing, China

## Abstract

High glucose- (HG-) induced cardiomyocyte injury is the leading cause of diabetic cardiomyopathy, which is associated with the induction of inflammatory responses and oxidative stress. TGR5 plays an important role in the regulation of glucose metabolism. However, whether TGR5 has cardioprotective effects against HG-induced cardiomyocyte injury is unknown. Neonatal mouse cardiomyocytes were isolated and incubated in a HG medium. Protein and mRNA expression was detected by western blotting and RT-PCR, respectively. Cell apoptosis was determined by Hoechst 33342 staining and flow cytometry. After treatment of cells with HG, TGR5-selective agonist INT-777 reduced the increase in expression of proinflammatory cytokines and NF-*κ*B, whereas pretreatment of cells with TGR5 shRNA significantly reduced the inhibitory effects of INT-777. We also found that INT-777 increased the protein expression of Nrf2 and HO-1. In the presence of TGR5 shRNA, the expression of Nrf2 and HO-1 was reduced, indicating that TGR5 may exert an antioxidant effect partially through the Nrf2/HO-1 pathway. Furthermore, INT-777 treatment inhibited HG-induced ROS production and apoptosis that were attenuated in the presence of TGR5 shRNA or ZnPP (HO-1 inhibitor). Activation of TGR5 has cardioprotective effects against HG-induced cardiomyocyte injury and could be a pharmacological target for the treatment of diabetic cardiomyopathy.

## 1. Introduction

Type 2 diabetes mellitus (T2DM) is a major health concern and burden on economies, which affected 2.8% of the world population in 2000 with morbidity estimated to reach 4.4% by 2030 [1]. T2DM is associated with many cardiovascular diseases. The cardiovascular complications are wide-ranging and due at least in part to chronic elevation of blood glucose levels. Although vascular damage is the most devastating complication of diabetes, hyperglycemia-induced myocardium injury in the absence of coronary artery disease and hypertension, termed as diabetic cardiomyopathy [2–4], has received increasing attention in particular [5, 6]. The cellular and molecular mechanisms underlying hyperglycemia-induced cardiac damage are complicated and multifactorial. Reactive oxygen species (ROS) elevation, inflammation, and apoptosis, which are caused by high blood glucose, are major precursors and contributors to the development of diabetic cardiomyopathy [7–9]. Such pathological changes induce diastolic function impairments, myocardial fibrosis, and cardiac complications [3, 6, 8, 9].

A member of the G protein-coupled receptor family, G protein-coupled bile acid receptor 1 (GPBAR1; also known as TGR5), has been recently identified as a drug target for diabetes treatment [10–12]. TGR5 is activated by bile acids and mediates the endocrine effects of bile acids on energy balance, inflammation, digestion, and sensation [13]. Bile acids have been reported to increase energy expenditure and prevent the development of obesity and insulin resistance, which are mediated by TGR5 [14]. A previous study has demonstrated that TGR5 in pancreatic cells regulates insulin secretion to maintain glucose homeostasis [12]. Based on these studies, TGR5 is thought to be a critical receptor for regulation of energy expenditure and a potential therapeutic target of diabetes. However, whether TGR5 activation prevents diabetes-associated cardiomyocyte injury is still unclear.

TGR5 expression has been found in cardiomyocytes and is associated with dysregulation of biliary fibrosis-induced cardiac energetics [15], suggesting that TGR5 plays an important role in cardiovascular homeostasis. Moreover, TGR5 activation reduces cytokine release and diminishes the inflammatory response [13, 16]. TGR5 is also linked to signaling pathways involved in cell survival and apoptosis [17]. Thus, we hypothesized that activation of TGR5 may be beneficial for protection against high glucose- (HG-) induced cardiomyocyte injury. Although a recent study showed that TGR5 activation improves myocardial adaptability to physiological, inotropic, and pressure-induced stress in mice [18], the role of TGR5 in HG-induced cardiomyocyte injury and the related molecular mechanisms are largely unknown.

## 2. Methods

### 2.1. Primary Mouse Cardiomyocyte Isolation, Culture, and Treatment

The protocol for the animal experiments was approved by the Experimental Animal Ethics Committee of Southwest Medical University. Animal handling and care were performed in strict compliance with the U.S. National Institutes of Health Guide for the Care and Use of Laboratory Animals (1996 revision). Primary cardiomyocytes were isolated from 20 to 30 neonatal Kunming mouse hearts, following a protocol reported previously [19, 20] with minor modifications. Less than 3-day-old neonatal mice were briefly rinsed in an antiseptic 75% alcohol solution. Mice were anaesthetized with 1% isoflurane and then euthanized by decapitation. Whole hearts were extracted from the mice using curved scissors and quickly transferred into a dish containing phosphate-buffered saline (PBS) without Ca^2+^ and Mg^2+^. Nonmyocardial tissues, such as lung tissues and large vessels, were removed. Neonatal mouse hearts were washed in PBS without Ca^2+^ and Mg^2+^ to remove blood. The neonatal mouse hearts were then transferred into another dish containing 0.25% trypsin-EDTA and incubated at 4°C overnight. The next day, the trypsin-EDTA solution was removed, and fetal bovine serum (FBS) was added. The hearts were then digested in a collagenase solution containing 0.5–1.0 mg/mL collagenase and 5 mg/mL albumin at 37°C. The digested tissues were dispersed by pipetting for 10 min, and the cell suspension was transferred into sterile centrifuge tubes. Fresh collagenase solution was added to the remaining tissues, and the above process was repeated two to four times until all tissues were digested and all cells were collected. After centrifugation, the harvested cells were resuspended in a 10 mL plating medium (DMEM supplemented with 10% FBS and 1% penicillin-streptomycin), plated in a 10 cm culture dish, and incubated at 37°C with 5% CO_2_ for 70 min. Unattached cardiomyocytes were collected from the 10 cm dish, resuspended in a fresh medium with 5-bromodeoxyuridine (0.1 mmol/L), and seeded in a 35 mm dish or 24-well culture plate coated with polylysine.

### 2.2. Gene Silencing by RNAi

TGR5 shRNA adenoviral particles were obtained from Hanbio Biotechnology (Shanghai, China), and Nrf2 shRNA lentiviral particles were obtained from Genechem Corporation (Shanghai, China). Experiments were performed in accordance with the manufacturer's instructions. Cells were infected with TGR5 or Nrf2 shRNA viral particles for 48 h before experiments.

### 2.3. Immunoblotting

Tissues or cells were washed twice with ice-cold PBS and lysed in lysis buffer (10 mM Tris-HCl, pH 8, 150 mM NaCl, 1% NP-40, 1 mM PMSF, and 10 mg/mL each leupeptin and aprotinin). The lysates (20 *μ*g protein) were separated by 10%–12% SDS-polyacrylamide gel electrophoresis and transferred onto a polyvinylidene fluoride membrane. The membrane was washed with Tris-buffered saline+Tween-20 (TBST), blocked with 5% skim milk powder in TBST for 3 h, and then incubated with the appropriate primary antibody at dilutions recommended by the supplier. Membranes were probed with a rabbit anti-phospho-I*κ*B*α* (Ser32) antibody (1 : 1000, Cell Signaling Technology, MA), mouse anti-NF-*κ*B p65 (L8F6) antibody (1 : 1000, Cell Signaling Technology), rabbit anti-Nrf2 (D1Z9C) antibody (1 : 1000, Cell Signaling Technology), rabbit anti-HO-1 antibody (1 : 1000, Cell Signaling Technology), or anti-TGR5 antibody (1 : 500, Santa Cruz Biotechnology, CA). Then, the primary antibodies were detected with goat anti-rabbit or anti-mouse IgG (1 : 1000, Beyotime, China) conjugated with horseradish peroxidase. Bands were visualized with enhanced chemiluminescence (Pierce, MA). Equal amounts of protein transferred onto the membranes were verified by immunoblotting for GAPDH (1 : 1000, Cell Signaling Technology) or histone H3 (1 : 500, Biogot Biotechnology CO, Atlanta, Georgia, USA).

### 2.4. RNA Extraction and PCR

Total RNA from cells and tissues was extracted by Trizol (Tiangen, Beijing, China) and used to synthesize cDNA that served as the template for amplification of TNF-*α*, IL-6, and IL-1*β* genes. Primers were as follows: TNF-*α* forward primer, 5′-GGCGGTGCCTATGTCTCA-3′ and TNF-*α* reverse primer, 5′-GGCAGCCTTGTCCCTTGA-3′ (363 bp); IL-6 forward primer, 5′-GCCTTCTTGGGACTGAT-3′ and IL-6 reverse primer, 5′-CTGGCTTTGTCTTTCTTGT-3′ (383 bp); IL-1*β* forward primer, 5′-CTCGTGCTGTCGGACCCAT-3′ and IL-1*β* reverse primer, 5′-CAGGCTTGTGCTCTGCTTGTGA-3′ (343 bp). The amplifications were performed using a reverse transcription-polymerase chain reaction (RT-PCR) kit (Tiangen). Mouse GAPDH was used as the endogenous control. The relative expression of target genes was normalized to GAPDH mRNA levels.

### 2.5. Hoechst 33342 Staining

Apoptotic cells were characterized by nuclear condensation of chromatin and/or nuclear fragmentation using a Hoechst 33342 staining kit (Solarbio, China), according to the manufacturer's instructions. In brief, after treatments, the cells were fixed with 1 mL staining buffer and then stained with 5 *μ*L Hoechst 33342 and 5 *μ*L PI. The cells were incubated at 4°C or in an ice bath for 20–30 min. After incubation, the cells were washed once with PBS and spotted onto slides for microscopy.

### 2.6. Flow Cytometric Analysis

Apoptosis and intracellular ROS levels were measured by flow cytometry [21]. To analyze ROS production, the cells were incubated with DCFH-DA (10 *μ*M) at 37°C for 20 min, and then the intracellular ROS level was determined by flow cytometry. For apoptosis analysis, cells were trypsinized, harvested, washed twice with cold PBS, and then centrifuged, followed by removal of the supernatant and resuspension in 1 mL of 1x binding buffer. The cells were gently vortexed, incubated for 10 min at room temperature while protected from light, and then stained with 5 *μ*L Annexin V-FITC. Then, the cells were stained with 5 *μ*L of a PI solution at room temperature for 5 min while protected from light. The cells were resuspended with 500 *μ*L PBS and vortexed gently. Cells were analyzed by flow cytometry within 1 h. FITC and PI fluorescence were excited by a laser source with a wavelength of 494 nm and detected by 520 and 636 nm band-pass filters, respectively.

### 2.7. Measurement of Cell Viability

Cell viability was determined using the conventional methylthiazolyl tetrazolium (MTT) reduction assay. After treatments, cell viability was measured at various time points. Twenty microliters of MTT (5 mg/mL) was added to each well, followed by incubation for 4 h at 37°C with CO_2_. The medium was then removed, and the formazan crystals were solubilized with DMSO. Absorbance was measured at 490 nm on a microplate reader (Bio-Rad, Hercules, CA).

### 2.8. Statistical Analysis

Data are expressed as the mean ± SD. All statistical analyses were performed by one-way analysis of variance (ANOVA) for repeated measures, and comparisons among groups were made by one-way ANOVA with the Student-Newman-Keuls test. A value of *P* < 0.05 was considered as significant.

## 3. Results

### 3.1. High Glucose Promotes the mRNA Expression of Proinflammatory Cytokines and Activation of NF-*κ*B Signaling in Mouse Cardiomyocytes

**Figure 1:**
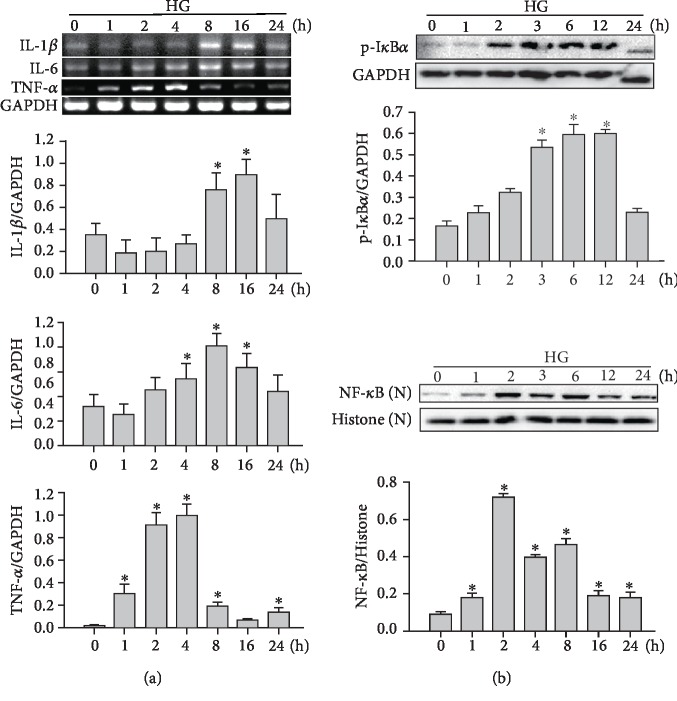
HG promotes mRNA expression of proinflammatory cytokines and activation of NF-*κ*B signaling in mouse cardiomyocytes. (a) HG-induced proinflammatory cytokine expression. Cardiomyocytes were treated with HG (33 mM) for the indicated times. IL-1*β*, IL-6, and TNF-*α* mRNA expression was determined by RT-PCR. (b) HG-induced activation of NF-*κ*B signaling. Cardiomyocytes were treated with HG (33 mM) for the indicated times. Proteins (20 *μ*g) from cell lysates or nuclear (N) fractions were subjected to immunoblotting. Data are expressed as the mean ± SD of three independent experiments. ^∗^*P* < 0.05*vs*. 0 h.

To determine the effects of HG on the inflammatory response in mouse cardiomyocytes, the mRNA expression of proinflammatory cytokines was measured. Cardiomyocytes were exposed to HG for 1, 2, 4, 8, 16, and 24 h. In cells treated with 33 mM HG, the mRNA expression of IL-1*β*, IL-6, and TNF-*α* was increased up to 16, 8, and 4 h, respectively, and decreased thereafter (Figure 1(a)). The NF-*κ*B signaling pathway was also analyzed. Cardiomyocytes were exposed to HG for 1, 2, 3, 6, 12, and 24 h. HG increased the protein expression of p-I*κ*B*α* and NF-*κ*B in cardiomyocytes. The protein expression of p-I*κ*B*α* and NF-*κ*B was increased up to 6 and 2 h, respectively, and decreased thereafter (Figure 1(b)). These data indicated that HG may induce the inflammatory response through activation of NF-*κ*B signaling in mouse cardiomyocytes.

### 3.2. Activation of TGR5 Reduces HG-Induced Increases in the Expression of Proinflammatory Cytokines and NF-*κ*B in Mouse Cardiomyocytes

**Figure 2:**
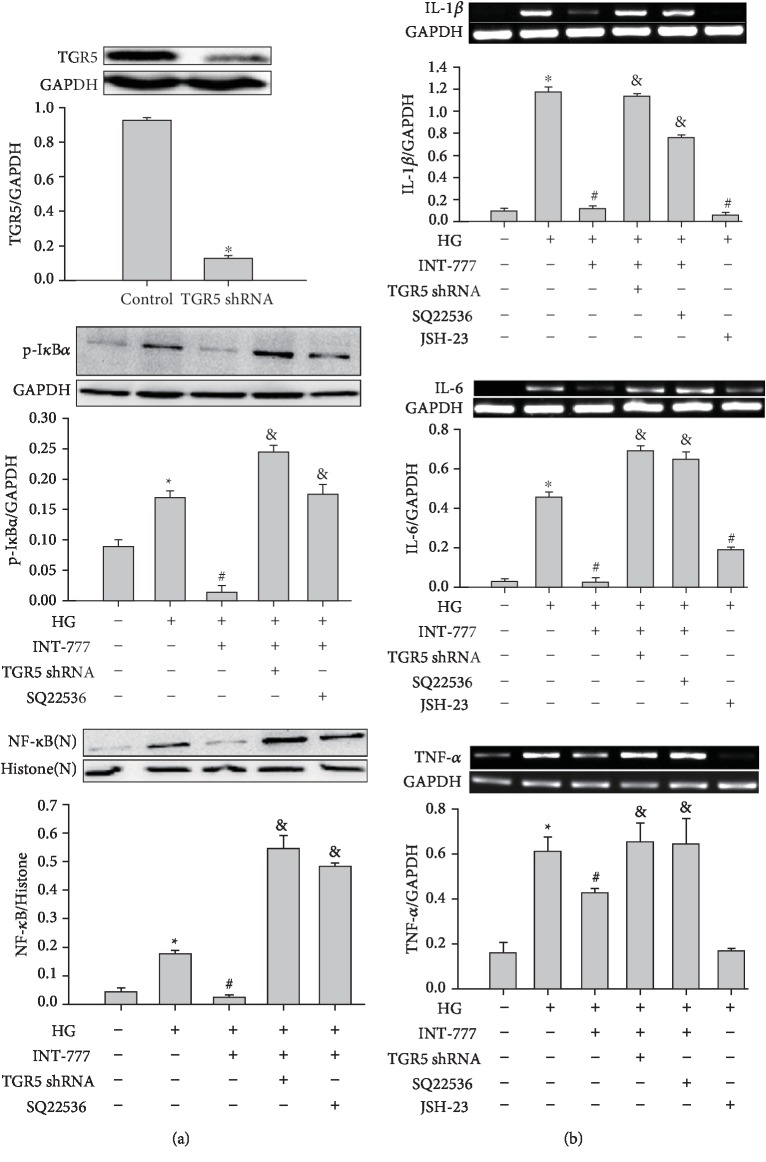
Activation of TGR5 reduces the HG-induced increase in the expression of proinflammatory cytokines and NF-*κ*B signaling in mouse cardiomyocytes. (a) Cells were infected with TGR5 shRNA adenoviral particles for 48 h or pretreated with 100 *μ*M SQ22536 (cAMP inhibitor) for 3 h and then treated with 30 *μ*M INT-777 (selective TGR5 agonist) for 3 h, followed by addition of HG to cardiomyocytes for a further 6 h. Proteins (20 *μ*g) from cell lysates or nuclear (N) fractions were subjected to immunoblotting. (b) Cells were infected with TGR5 shRNA adenoviral particles for 48 h or pretreated with 100 *μ*M SQ22536 for 3 h and then treated with 30 *μ*M INT-777 for 3 h or 30 *μ*M JSH-23 (NF-*κ*B inhibitor) for 1 h, followed by exposure to HG for a further 2 h (TNF-*α*) or 8 h (IL-1*β* and IL-6). IL-1*β*, IL-6, and TNF-*α* mRNA expression was determined by RT-PCR. Data are expressed as the mean ± SD of three independent experiments. ^∗^*P* < 0.05*vs*. control; ^#^*P* < 0.05*vs*. HG; ^&^*P* < 0.05*vs*. HG+INT-777.

To determine the effect of TGR5 on the inflammatory response, cardiomyocytes were infected with TGR5 shRNA adenoviral particles for 24 h or pretreated with 100 *μ*M SQ22536 (cAMP inhibitor) for 3 h and then treated with 30 *μ*M INT-777 (selective TGR5 agonist) for 3 h, followed by exposure to HG for 6 h. As shown by western blotting, pretreatment of cardiomyocytes with INT-777 inhibited HG-induced protein expression of p-I*κ*B*α*, whereas the inhibitory effect of INT-777 was attenuated by TGR5 shRNA or SQ22536 (Figure 2(a)). Similarly, INT-777 inhibited HG-induced protein expression of NF-*κ*B, and TGR5 shRNA or SQ22536 reduced the inhibitory effects of INT-777 (Figure 2(a)). Furthermore, INT-777 or JSH-23 (NF-*κ*B inhibitor) suppressed the HG-induced mRNA expression of IL-1*β*, IL-6, and TNF-*α*. Treatment with TGR5 shRNA or SQ22536 ameliorated the inhibitory effects of INT-777 on the mRNA expression of IL-1*β*, IL-6, and TNF-*α* (Figure 2(b)). These results suggested that the NF-*κ*B signaling pathway was involved in TGR5-mediated cardioprotection.

### 3.3. Activation of TGR5 Induces the Nrf2/HO-1 Pathway in Mouse Cardiomyocytes

**Figure 3:**
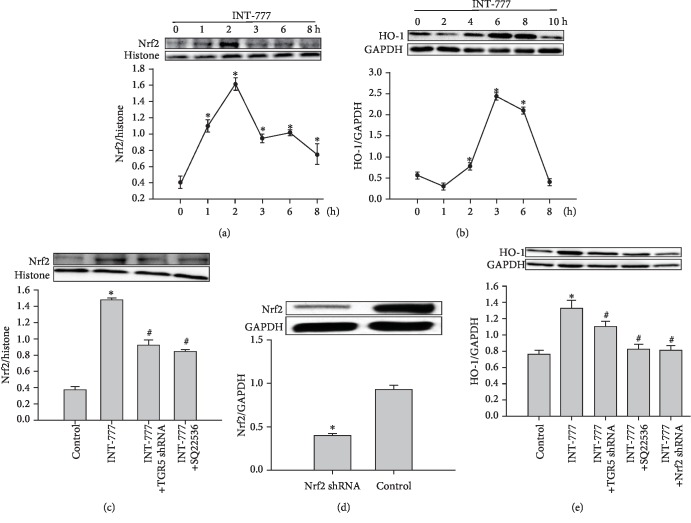
Activation of TGR5 induces the Nrf2/HO-1 pathway in mouse cardiomyocytes. (a) Nuclear extracts were prepared from cardiomyocytes treated with INT-777 (30 *μ*M) at the indicated time points to detect Nrf2 protein by immunoblotting. (b) Cells were treated with 30 *μ*M INT-777 for the indicated times to detect HO-1 by immunoblotting. (c) Cells were infected with TGR5 shRNA adenoviral particles for 48 h or pretreated with 100 *μ*M SQ22536 (cAMP Inhibitor) for 3 h and then treated 30 *μ*M INT-777 for 2 h. Proteins (20 *μ*g) from nuclear (N) fractions were subjected to immunoblotting. (d) Cells were infected with Nrf2 shRNA lentiviral particles for 48 h. Proteins from cell lysates were subjected to immunoblotting. (e) Cells were infected with TGR5 or Nrf2 shRNA viral particles for 48 h or pretreated with 100 *μ*M SQ22536 for 3 h and then treated with 30 *μ*M INT-777 for 6 h. Proteins from cell lysates were subjected to immunoblotting. Data are expressed as the mean ± SD of three independent experiments. ^∗^*P* < 0.05*vs*. 0 h or control; ^#^*P* < 0.05*vs*. HG.

The Nrf2/HO-1 signaling pathway is an important regulator of oxidative stress. Therefore, we determined whether activation of TGR5 induces the Nrf2/HO-1 axis in cardiomyocytes. TGR5 agonist INT-777 increased the protein expression of Nrf2 and HO-1 in cardiomyocytes up to 2 and 6 h, respectively, which was decreased thereafter (Figures 3(a) and 3(b)). The increase in protein expression of Nrf2 by INT-777 treatment was reduced after pretreatment with TGR5 shRNA or SQ22536 (Figure 3(c)). Next, cells were infected with either control or Nrf2 shRNA. Nrf2 shRNA significantly abated Nrf2 protein expression compared with the control (Figure 3(d)). Similarly, INT-777-induced HO-1 expression was also reduced by TGR5 shRNA, SQ22536, or Nrf2 shRNA (Figure 3(e)). These findings indicated that activation of TGR5 may have antioxidant effects partially mediated by inducing the Nrf2/HO-1 pathway.

### 3.4. Activation of TGR5 Alleviates HG-Induced Oxidative Stress in Mouse Cardiomyocytes

**Figure 4:**
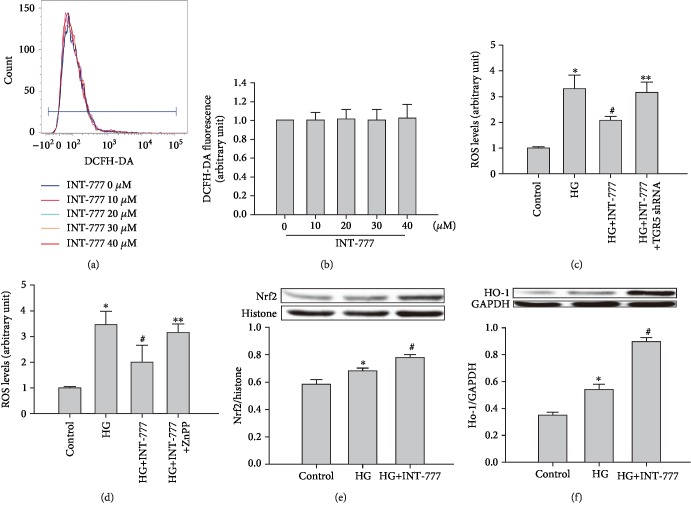
Effect of TGR5 activation on HG-induced oxidative stress in mouse cardiomyocytes. (a, b) ROS production was detected by flow cytometry with DCFH-DA staining at 20 min after INT-777 treatment. ROS levels were calculated as fold changes relative to no treatment (0 *μ*M). (c, d) Changes in ROS production were detected by flow cytometry with DCFH-DA staining. Cells were infected with TGR5 shRNA adenoviral particles for 48 h, or cells were pretreated with 10 *μ*M ZnPP for 1 h and then treated with INT-777 for 3 h, followed by addition of HG to cells for a further 12 h. ROS levels were calculated as fold changes relative to the control. (e, f) Immunoblotting for the expression of Nrf2 and HO-1 in cells. Cells were treated with INT-777 for 3 h, followed by addition of HG to cells for a further 12 h. Data are expressed as the mean ± SD of three independent experiments. ^∗^*P* < 0.05*vs*. control; ^#^*P* < 0.05*vs*. HG; ^∗∗^*P* < 0.05*vs*. HG+INT-777.

To examine the benefit of TGR5 against HG insult, oxidative stress and Nrf2/HO-1 axis (an antioxidant response system) were examined in primary neonatal mouse cardiomyocytes. Flow cytometry showed that INT-777 alone at various concentrations did not induce ROS production (Figures 4(a) and 4(b)). However, when cells were treated with HG, the intracellular ROS level was increased. INT-777 significantly reduced the level of ROS in HG-treated cells, whereas the effect of INT-777 was abolished in the presence of TGR5 shRNA or the HO-1 inhibitor ZnPP (Figures 4(c) and 4(d)). Immunoblotting data showed that Nrf2 and HO-1 were increased in HG condition compared with the control group (Figures 4(e) and 4(f)), while INT-777 treatment further increased the expression of Nrf2 and HO-1 significantly compared with the HG group (Figures 4(e) and 4(f)).

### 3.5. Activation of TGR5 Attenuates HG-Induced Apoptosis in Mouse Cardiomyocytes

**Figure 5:**
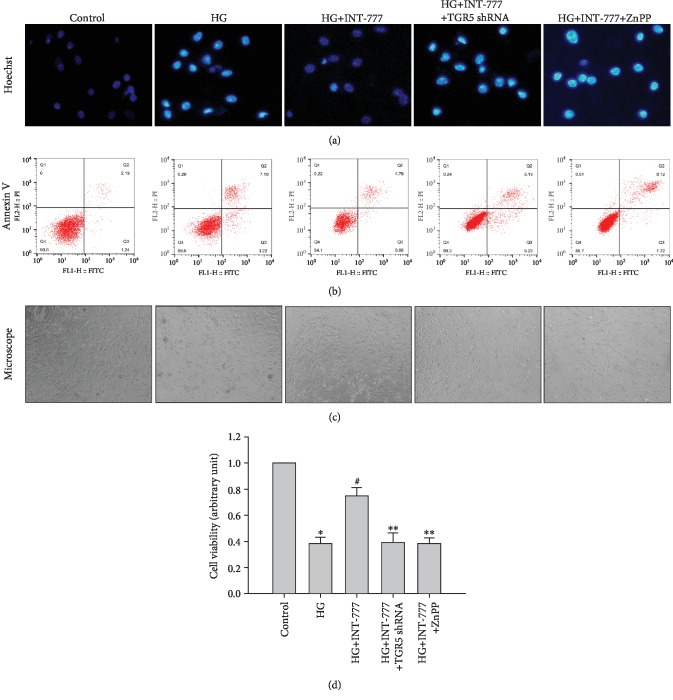
Activation of TGR5 attenuates HG-induced apoptosis in mouse cardiomyocytes. (a) Cells were infected with TGR5 shRNA adenoviral particles for 48 h or pretreated with 10 *μ*M ZnPP for 1 h and then treated with 30 *μ*M INT-777 for 3 h, followed by addition of HG to cardiomyocytes for a further 24 h. Cells with condensed or fragmented nuclei were defined as apoptotic cells. (b) Cells were infected with TGR5 shRNA adenoviral particles for 48 h or pretreated with 10 *μ*M ZnPP for 1 h and then treated with 30 *μ*M INT-777 for 3 h, followed by addition of HG to cardiomyocytes for a further 24 h. Cells were stained with FITC-labeled Annexin V and PI, and flow cytometry was used to detect apoptotic cells. (c) Microscopy was used to observe the morphology of mouse cardiomyocytes treated as described in (a) and (b). (d) Cell viability was measured by MTT assays. ^∗^*P* < 0.05*vs*. control; ^#^*P* < 0.05*vs*. HG; ^∗∗^*P* < 0.05*vs*. HG+INT-777.

To investigate the effect of TGR5 on HG-induced apoptosis, we employed Hoechst 33342 staining and flow cytometry to measure apoptosis after treatment with HG, INT-777, TGR5 shRNA, and ZnPP. As shown in Figure 5(a), HG increased nuclear condensation that was inhibited by INT-777. Pretreatment with TGR5 shRNA or ZnPP mitigated the protective effect of INT-777 against HG-induced nuclear condensation. Similarly, INT-777 reduced HG-induced apoptosis, whereas the protective effect of INT-777 was ameliorated by TGR5 shRNA or ZnPP (Figure 5(b)). Microscopic examination revealed results similar to those of Hoechst 33342 staining and flow cytometry (Figure 5(c)). Furthermore, the effect of TGR5 was analyzed by MTT assays. Cell death was significantly increased under the HG condition. Cardiomyocytes treated with INT-777 showed suppression of cell death induced by HG. Cell death was increased in the presence of TGR5 shRNA or ZnPP (Figure 5(d)). These data support the notion that activation of TGR5 reduces HG-induced apoptosis partially through the Nrf2/HO-1 pathway.

## 4. Discussion

TGR5 is a G protein-coupled receptor that plays a key role in the physiological activities of bile acid [13]. Previous studies have indicated that TGR5 may be a novel target to regulate glucose metabolism for diabetes therapy. The TGR5 ligand (oleanolic acid) shows significant blood glucose-lowering and weight-losing effects in diabetic animal models induced by STZ [22, 23] and enhances glucose tolerance [10]. TGR5 activation by oleanolic acid induces glucagon-like peptide-1 production and secretion that improve liver and pancreas functions [24, 25]. However, the effects of TGR5 on HG-induced cardiomyocyte damage are unclear. Our data are consistent with the hypothesis that TGR5 activation protects against HG-induced cardiomyocyte damage by suppressing inflammatory cytokines and ROS overproduction and that TGR5 may be a pharmacological target for the treatment of HG-induced disorders.

Myocardial injury involved in T2DM is mainly due to direct metabolic damage of cardiomyocytes by hyperglycemia. Inflammatory responses and oxidative stress are implicated in the mechanism, and inhibiting the inflammatory response and oxidative stress is useful for diabetic cardiomyopathy. For example, K_ATP_ channel opening protects H9c2 cardiac cells against HG-induced injury by inhibiting ROS and inflammation [26]. Suppressing the inflammatory response and oxidative stress by kaempferol also attenuates hyperglycemia-induced cardiac damage [27]. Moreover, aza resveratrol-chalcone derivative 6b protects mice against diabetic cardiomyopathy by alleviating inflammation and oxidative stress [28]. Therefore, we focused on the effect of TGR5 on the inflammatory response and oxidative stress in cardiomyocytes.

Activation of TGR5 results in the cascade of the adenylyl cyclase/cAMP signaling pathway [13]. The cAMP signaling pathway mediates inhibition of TGR5 binding to NF-*κ*B and lowers proinflammatory cytokines IL-1*α*, IL-1*β*, IL-6, and TNF-*α* [16, 29]. Because of reports indicating an important role of TGR5 in the regulation of inflammation [13, 16, 17], we considered that TGR5 may protect against HG-induced cardiomyocyte inflammation. NF-*κ*B has been identified as a key regulator of the inflammatory response, which transcriptionally activates proinflammatory cytokine genes such as IL-1*β*, IL-6, and TNF-*α* [30, 31]. Under inactivated conditions, NF-*κ*B is localized in the cytoplasm by its inhibitory molecule (I*κ*B). Upon stimulation, I*κ*B kinase phosphorylates I*κ*B*α*. As a result, NF-*κ*B dissociates from the complex and translocates into the nucleus to regulate target genes [32]. In our study, HG increased the translocation of NF-*κ*B into the nucleus by increasing the phosphorylation of I*κ*B*α*. Moreover, treatment of cardiomyocytes with JSH-23 (an NF-*κ*B inhibitor) greatly suppressed the HG-induced mRNA expression of proinflammatory cytokines, including IL-1*β*, IL-6, and TNF-*α*, suggesting involvement of the NF-*κ*B pathway in HG-induced expression of proinflammatory cytokines. Next, we examined whether activation of TGR5 reduced proinflammatory cytokines through NF-*κ*B pathways. The TGR5-selective ligand INT-777 inhibited the HG-induced phosphorylation of I*κ*B*α* and nuclear translocation of NF-*κ*B in cardiomyocytes. The inhibitory effects of INT-777 were significantly attenuated by knockdown of TGR5 or treatment with SQ22536 (a cAMP inhibitor). These results suggested that activation of TGR5 under HG conditions exerts anti-inflammatory effects by suppressing the NF-*κ*B pathway in cardiomyocytes.

In addition to inflammation, hyperglycemia-induced ROS overproduction contributes to the development of cardiac complications in diabetic patients. As a cytoprotective enzyme, HO-1 has critical antioxidant functions [33]. Nrf2, a transcription factor, regulates HO-1 promoter activity and induces HO-1 expression [34]. It has been reported that the Nrf2/HO-1 pathway is involved in the pathophysiological processes of diabetes and cardiac complications [35–37]. Therefore, we speculated that the Nrf2/HO-1 pathway may be involved in the cytoprotection of TGR5. Our study showed that INT-777 induced Nrf2 nuclear localization and upregulated HO-1 expression. Furthermore, increased HO-1 protein expression induced by INT-777 was inhibited by Nrf2 shRNA. TGR5 shRNA also attenuated Nrf2 nuclear localization and the subsequent upregulation of HO-1 expression. Under HG conditions, treatment with INT-777 also increased the protein expression of Nrf2 and HO-1 in cardiomyocytes. Notably, the increase in HO-1 expression induced by INT-777 was not completely eliminated by TGR5 shRNA in this study. Therefore, we believe that Nrf2/HO-1 signaling may be partially mediated by TGR5, indicating that activation of TGR5 may exert antioxidant effects partially through the Nrf2/HO-1 pathway.

The effect of TGR5 activation on ROS generation remains controversial. Previous studies have shown that TGR5 is essential for bile acid-dependent cholangiocyte proliferation by increasing reactive oxygen species [38], and TGR5 mediates taurodeoxycholic acid-induced H_2_O_2_ production in human Barrett's and oesophageal adenocarcinoma cells [39]. However, Wang et al. [40] showed that TGR5 activated by INT-777 decreases oxidative stress and increases fatty acid *β*-oxidation in human podocytes treated with HG. Additionally, INT-777 extenuates pancreatic acinar cell necrosis by inhibiting ROS production and the NLRP3 inflammasome pathway [41]. Moreover, lithocholic acid (a natural agonist of TGR5) does not affect HG-induced elevation of ROS production in H9c2 cells [42]. These inconsistent observations may be due to different agonists, cell types, and experimental conditions. In the present study, we found that ROS was not induced by treatment with INT-777 at various concentrations, and INT-777 decreased HG-induced ROS production in cardiomyocytes. The antioxidant effect of INT-777 was abrogated by TGR5 shRNA or the HO-1 inhibitor ZnPP. Furthermore, HG-induced cell apoptosis was reduced by INT-777. Treatment of cells with TGR5 shRNA or ZnPP also attenuated the protective effect of INT-777.

In addition to the heart, TGR5 is also expressed in other organs of the body, especially the gallbladder. Previous studies have shown that activation of TGR5 exerts antidiabetic effects but causes gallbladder filling that may induce unwanted toxic effects on the gallbladder [43, 44]. Accordingly, local activation of TGR5 needs to be applied in the future.

## 5. Conclusions

**Figure 6:**
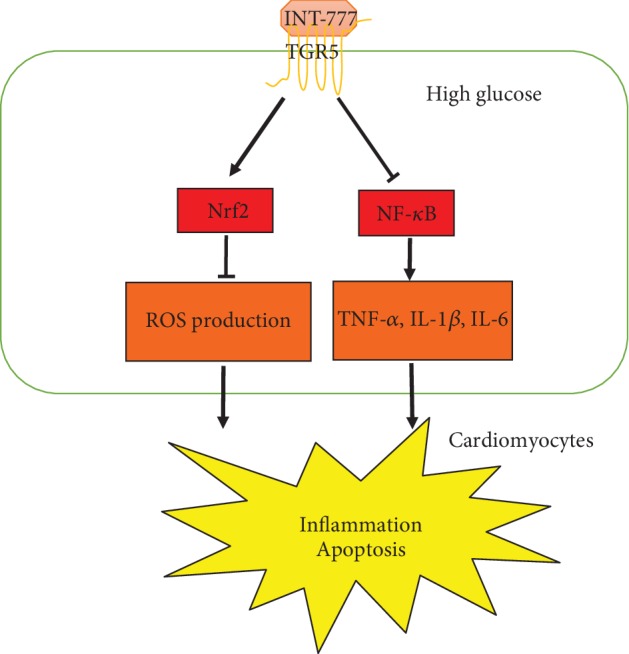
Schematic illustration of the preventive effect of TGR5 against HG-induced injury in cardiomyocytes. HG induces ROS production and expression of proinflammatory cytokine, such as IL-1*β*, IL-6, and TNF-*α*, resulting in inflammation and apoptosis. Activation of TGR5 attenuates HG-induced cardiomyocyte injury partially by inhibiting the NF-*κ*B pathway and activating the Nrf2 pathway.

Activation of TGR5 protected against HG-induced cardiomyocyte injury by suppressing inflammation and apoptosis partially through inhibiting the NF-*κ*B pathway and activating the Nrf2 pathway (Figure 6). Therefore, TGR5 could be a pharmacological target for the treatment of diabetic cardiomyopathy.

## Data Availability

The data used to support the findings of this study are included within the article.
